# Cyclic Amp-Dependent Resuscitation of Dormant Mycobacteria by Exogenous Free Fatty Acids

**DOI:** 10.1371/journal.pone.0082914

**Published:** 2013-12-23

**Authors:** Margarita Shleeva, Anna Goncharenko, Yuliya Kudykina, Danielle Young, Michael Young, Arseny Kaprelyants

**Affiliations:** 1 Bach Institute of Biochemistry Russian Academy of Sciences, Moscow, Russia; 2 Institute of Biological, Environmental and Rural Sciences, Aberystwyth University, Aberystwyth, United Kingdom; Institut Pasteur, France

## Abstract

One third of the world population carries a latent tuberculosis (TB) infection, which may reactivate leading to active disease. Although TB latency has been known for many years it remains poorly understood. In particular, substances of host origin, which may induce the resuscitation of dormant mycobacteria, have not yet been described. *In vitro* models of dormant (“non-culturable”) cells of *Mycobacterium smegmatis* (mc^2^155) and *Mycobacterium tuberculosis* H37Rv were used. We found that the resuscitation of dormant *M. smegmatis* and *M. tuberculosis* cells in liquid medium was stimulated by adding free unsaturated fatty acids (FA), including arachidonic acid, at concentrations of 1.6–10 µM. FA addition enhanced cAMP levels in reactivating *M. smegmatis* cells and exogenously added cAMP (3–10 mM) or dibutyryl-cAMP (0.5–1 mM) substituted for FA, causing resuscitation of *M. smegmatis* and *M. tuberculosis* dormant cells. A *M. smegmatis* null-mutant lacking MSMEG_4279, which encodes a FA-activated adenylyl cyclase (AC), could not be resuscitated by FA but it was resuscitated by cAMP. *M. smegmatis* and *M. tuberculosis* cells hyper-expressing AC were unable to form non-culturable cells and a specific inhibitor of AC (8-bromo-cAMP) prevented FA-dependent resuscitation. RT-PCR analysis revealed that *rpfA* (coding for resuscitation promoting factor A) is up-regulated in *M. smegmatis* in the beginning of exponential growth following the cAMP increase in lag phase caused by FA-induced cell activation. A specific Rpf inhibitor (4-benzoyl-2-nitrophenylthiocyanate) suppressed FA-induced resuscitation. We propose a novel pathway for the resuscitation of dormant mycobacteria involving the activation of adenylyl cyclase MSMEG_4279 by FAs resulted in activation of cellular metabolism followed later by increase of RpfA activity which stimulates cell multiplication in exponential phase. The study reveals a probable role for lipids of host origin in the resuscitation of dormant mycobacteria, which may function during the reactivation of latent TB.

## Introduction

Tuberculosis (TB) latency is an intriguing phenomenon with important medical significance as one third of the global population is latently infected by the causative agent, *Mycobacterium tuberculosis*. Clinical and epidemiological studies have provided evidence of endogenous reactivation of *M. tuberculosis* after more than three decades of latent TB infection [1]. Although the mechanistic basis of TB latency is not well understood, the persistence of quiescent or dormant mycobacterial cells that act as a reservoir for subsequent reactivation TB is generally accepted [2]. Experimentally, two types of dormant cells could be considered. Firstly they may have lowered metabolic activity and remain culturable. This is exemplified Wayne’s oxygen-limited model of TB dormancy [3]. Secondly, cells may become profoundly dormant. In this case, they lose the ability to form colonies on solid media but they can resuscitate in liquid media either spontaneously, or upon addition of reactivation factors [4]. The second type of dormancy more closely reflects the situation *in viv*o, since *M. tuberculosis* cells isolated from animal organs exhibit a “non-culturable” (NC) phenotype [5], [6]. The transition of viable mycobacteria to the NC state and their exit from it are of cardinal importance for understanding the phenomenon of TB latency. Although some progress has been made recently, the molecular mechanisms that underlie these processes still remain obscure.

Resuscitation is a complex process, during which many reactions and pathways must be switched on in a temporally controlled and coordinated manner. A problem of particular interest is the initial step in the reactivation pathway, which triggers a cascade of enzymatic processes culminating in the formation of fully active, viable cells. Resuscitation of bacterial endospores is comparatively well understood. Simple metabolites like alanine & adenosine, (termed germinants) and also muropeptides (see below) bind to specific receptors stimulating ion transport which, in turn, activates lytic enzymes in the spore envelope that provoke its destruction [7]. Eventually, the germinating endospore begins to exchange intracellular materials with the external environment and metabolic activity resumes.

The Rpf proteins, which are believed to have muralytic activity, are widely distributed throughout the actinobacteria, including *M. tuberculosis*, and they are implicated in the resuscitation of dormant forms of these organisms [8]. There may be parallels between the initial steps of endospore germination in Bacilli and the resuscitation of dormant Actinobacteria. The recent finding that muropeptides (products released from cell walls by the activity of muralytic enzymes) germinate *Bacillus subtilis* spores [9] led to the suggestion that the Rpf proteins may release muropeptides in the surrounding medium and that they may play a signaling role for triggering the onset of resuscitation [10], [11], [12]. However, neither the release of muropeptides following the action of Rpf on the actinobacterial cell wall, nor the resuscitation activity of any individual muropeptides has been reported to date. Furthermore, any Rpf-dependent mechanism of resuscitation *in vivo* must of necessity rely on the presence of some residual metabolically active bacteria within the dormant cell population because both the Rpf proteins themselves and the muropeptides that may be released by their enzymatic activity are of bacterial origin. Moreover, the numbers of dormant mycobacteria in their animal or human hosts could be very low [13].

For these reasons, we suspected that the initial resuscitation stimulus is probably of host origin. One potential candidate would be lipids that are present in spent culture medium. Indeed, when phospholipids were added to agar plates the number of *M. tuberculosis* colonies recovered from starved cultures was increased [14]. In the present study we therefore investigated the possible role of lipid substances in the resuscitation of dormant bacteria using well-characterized models of NC mycobacterial cells.

We have discovered a new resuscitation mechanism involving the stimulation of adenylyl cyclase activity by unsaturated fatty acids (FA), which causes the resumption of the metabolic activity of NC/dormant cells. Rpf also plays a role in this resuscitation mechanism, presumably by remodeling of “dormant” peptidoglycan via its inferred lytic transglycosylase activity.

## Materials and Methods

### Bacterial Strains, Growth Media and Culture Conditions

This work was carried out using as wild type *Mycobacterium smegmatis* mc^2^155 (ATCC 700084). A mutant denoted ΔAC, which lacks the MSMEG_4279 gene encoding an adenylyl cyclase (AC) was constructed as detailed below. Derivatives of both strains harbouring plasmid pMind-AC (see below) were also employed. Hygromycin was added to the growth media at a concentration 50 µg/ml for plasmid-containing strains of *M. smegmatis.* For construction and growth of the ΔAC mutant kanamycin was used at a concentration 10 µg/ml. *E. coli* strain BMH 71–18 *mutS* was employed for cloning. All strains were routinely maintained on Nutrient Broth E (NBE) medium (HiMedia, India) supplemented with ampicillin (50 µg/ml) and/or kanamycin (10–50 µg/ml), as appropriate.

*M. smegmatis* strains were routinely grown for 24–30 h at 37°C on an orbital shaker (200 rpm) in a 150 ml conical flask containing 20 ml NBE medium to which 0.05% (v/v) Tween 80 was added.

**Figure 1:**
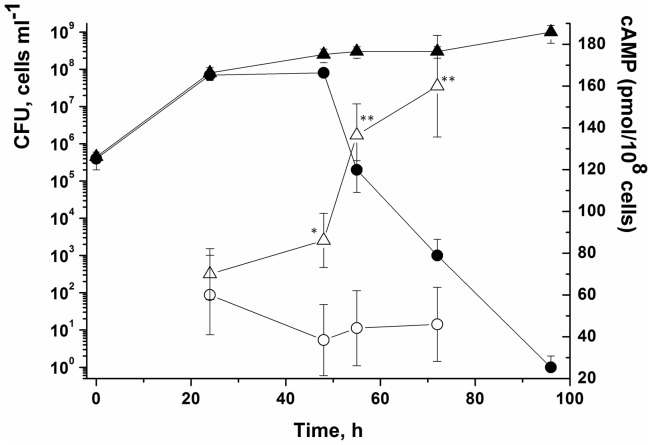
Hyper-expression of the MSMEG_4279 adenylyl cyclase abolishes the transition of *M. smegmatis* cells to the NC state. *M. smegmatis* cells were inoculated into modified (lacking potassium ions) Hartman’s–de Bont (mHdeB) medium and incubated with strong aeration 200 rpm) at 37°C. Strains harbouring pMind (empty plasmid vector) or pMindAc (adenylyl cyclase hyper-expression plasmid) are denoted with circles and triangles, respectively. Samples were withdrawn periodically for CFU determination (closed symbols) and intracellular cAMP content (open symbols). This experiment was repeated three times with similar results; the error bars represent the standard error of the mean. Asterisks indicate significant difference in cAMP concentration between pMindAC cells and pMind cells by Student’s t-test.

For the production of populations of NC cells of *M. smegmatis*, a modified (i.e. potassium-limited) form of Hartman’s–de Bont medium (mHdeB) was employed [15]. It contains (per liter) 11.8 g Na_2_HPO_4_·12 H_2_O, 1.1 g citric acid, 20 g (NH_4_)_2_SO_4_, 30 ml glycerol, 0.05% Tween 80, 0.5% BSA (fraction V, Cohn Analog; Sigma-Aldrich catalogue number A1470) and 10 ml of a microelement solution. One liter of the microelement solution contains 1 g EDTA, 10 g MgCl_2_·6 H_2_O, 0.1 g CaCl_2_·2 H_2_O, 0.04 g CoCl_2_·6 H_2_O, 0.1 g MnCl_2_·2 H_2_O, 0.02 g Na_2_MoO_4_·2 H_2_O, 0.2 g ZnSO_4_·7 H_2_O, 0.02 g CuSO_4_·5 H_2_O and 0.5 g FeSO_4_·7 H_2_O. A 1-ml inoculum from a fresh overnight culture in NBE was inoculated into a 300 ml conical flask containing 100 ml (mHdeB) medium. Growth proceeded for 72–76 h at 37°C on an orbital shaker (200 rpm) after which time the entire cell population had become NC (i.e. zero CFU when plated on NBE agar). A typical growth curve for *M. smegmatis* incubated under these conditions is shown later (see Figure 1).

For the production of populations of NC cells of *M. tuberculosis* strain H37Rv under gradual acidification in stationary phase [16], bacteria were initially grown for 12–15 d in 50 ml of unmodified Sauton’s medium supplemented with 0.05%Tween-80 and ADC on an orbital shaker (200 rpm) in a 150 ml conical flask. The culture, grown in the above-described medium, served as an inoculum for the production of NC cell populations. A 1-ml sample was introduced into 200 ml modified Sauton’s medium in a 650-ml conical flask and incubated at 37°C with shaking for up to 40–60 d. The pH value was measured periodically and when the medium in these post-stationary phase cultures reached pH6.0–6.2, the cultures were transferred to capped plastic tubes (50 ml) and 3-(N-morpholino)-propanesulfonic acid (MOPS) was added to a final concentration of 20 mM to prevent further acidification of the spent medium during long-term storage. Incubation was continued under static conditions (i.e. without agitation) at room temperature for up to 180 d post-inoculation. Cell populations became NC 60–70 d post-inoculation (CFU = 0). NC cells from 100–120 d old cultures were used for resuscitation experiments [16]. Before resuscitation, dormant cells were repeatedly passed through a gauge 21 needle to disrupt aggregates.

The resuscitation of NC cells of *M. smegmatis* and *M. tuberculosis* was accomplished using reactivation medium. This is twice diluted Sauton’s medium [17] supplemented with: 0.6% glycerol and 0.025% yeast extract (LabM) as well as 0.025% tyloxapol for *M. smegmatis*; 0.6% glycerol, 0.2% glucose, 0.085% NaCl and 0.025% tyloxapol for *M. tuberculosis*.

### Viability Estimation

Bacterial suspensions were serially diluted in fresh Sauton’s medium, and then three replicate 100-µl samples from each dilution were spotted on NBE agar. Plates were incubated at 37°C for 5 d then the number of colony forming units (CFU) was counted. The limit of detection was 5·10^0^ CFU/ml.

### Resuscitation of NC Cells

Resuscitation was performed by incubating NC cells in a liquid reactivation medium in one of two formats.

For MPN format, 48-well plastic plates (Corning, USA) were used; each well contained 1 ml reactivation medium. Some wells were supplemented with test compounds (e.g. free fatty acids, phospholipid liposomes or other substances of lipid nature) at various concentrations. Serially diluted *M. smegmatis* NC cells were added to three replicate wells. Plates were incubated at 37°C with agitation at 100 rpm for 10–14 d and the number of wells with visible bacterial growth was scored. Most probable number (MPN) values were determined using standard statistical tables [18]. In some experiments an RPF inhibitor was employed [19]; it was added at the start of the resuscitation period and every 2 days thereafter.

For batch format, the *M. smegmatis* and *M. tuberculosis* NC cells obtained as above were washed three times in 20–30 ml reactivation medium containing 0.025% tyloxapol and then resuspended in 20 ml reactivation medium in a 150 ml flask to give an initial OD_600_ = 0.1–0.3. Incubation was at 37°C for 6–11 d for *M. smegmatis* and for 20–25 d for *M. tuberculosis* with agitation at 100–120 rpm and cultures were sampled periodically for cell density measurement (OD_600_). In some experiments samples were plated on NBE agar.

### Metabolic Activity Estimation

The metabolic activity of cell suspensions was determined by monitoring the incorporation of ^3^H-uracil. Samples of cell suspensions (1 ml) were incubated with 1 µl [5,6-^3^H] uracil (10 µCi; 0.2 µmol) in 50% ethanol and incubated for 4 h at 37°C with agitation (45–60 rpm). The cells were then harvested on glass fibre filters (Whatman GFC), washed with 4 ml 10% trichloroacetic acid followed by 4 ml absolute ethanol. Air-dried filters were placed in scintillation liquid and the radioactivity incorporated was measured with a Beckman Coulter (United States) LS6500 scintillation counter.

### cAMP Determination

To determine intracellular levels of cAMP, samples of cell suspensions containing ca. 10^8^ cells were centrifuged (3000 g). The bacterial pellet was suspended in 1 ml 0.1 M HCl and the cells were disrupted with zirconia beads (0.1 mm,) using a mini Bead Beater (BioSpec Products, USA). Beads and bacterial debris were removed by centrifugation and the supernatants were used for cAMP estimation using a direct immunoassay kit (BioVision).

### RNA Isolation

RNA was extracted during the incubation of NC cells in reactivation medium (flask format) in the presence or absence of oleic acid. For each time point, 30-ml culture samples were employed from three independent experiments. Cells were harvested by centrifugation (4000 g, 10 min) and 1 ml Trizol reagent was added to the pellets. Cells were disrupted using zirconia beads (0.1 mm) in mini Bead Beater (BioSpec Products, USA). After centrifugation to remove particulates the supernatant was extracted once with chloroform. Nucleic acids were then precipitated with isopropanol, harvested by centrifugation, washed with 70% ethanol and re-dissolved in nuclease-free water (Promega, USA) containing RNAsin ribonuclease inhibitor (Promega, USA). RNA was then isolated using an RNeasy Mini kit (Qiagen). Each RNA sample was finally treated with RNase-free DNase1 (Ambion), which was then heat-inactivated according to the kit protocol. RNA was quantified using a Nanodrop ND1000 Spectrophotometer (Thermo Scientific).

### Quantitative Real-time PCR

For qRT-PCR, the iScript One-Step RT-PCR kit (BioRad) was employed with SYBR Green. Each reaction contained 50 ng RNA and 20 µMol each of the paired gene-specific primers shown in Figure S1. All primers were optimized for annealing temperature to ensure that only a single product of the correct size was amplified. The initial cDNA synthesis step was carried out at 50°C for 10 min after which the product was denatured for 5 min at 94°C. DNA amplification was for 40 cycles of 30 s at 94°C, then 30 s at 55°C and then 1 min at 72°C. The quality of each PCR product was verified by melt curve analysis. The PCR cycle at which the amplification threshold was attained was converted to copy number using standard curves prepared with *M. smegmatis* genomic DNA (1 pg *M. smegmatis* DNA corresponds to ca. 133 genome equivalents).

### DNA Manipulations

The pMind-AC (tetracycline-inducible) expression plasmid was constructed as follows. The MSMEG_4279 coding sequence together with 83 nt upstream and 77 nt downstream was amplified from *M. smegmatis* mc^2^155 genomic DNA using primers Up pMind**-**AC and Low pMind**-**AC (Figure S1). The 1252 bp amplification product was first cloned into the pGEM-T vector (Promega) and then sub-cloned as a 1250 bp *Bam*HI - *Spe*I fragment in the pMind expression vector [20]. Cells of *M. smegmatis* and *M. tuberculosis* were transformed with the pMind vector by electroporation.

To inactivate the MSMEG_4279 adenylyl cyclase gene we replaced ca. 900 bp of the coding sequence with a kanamycin-resistance cassette derived from plasmid pHP45 Ω-Km. This was accomplished as follows. Primers Up Δ*ac*/L & Low Δ*ac*/L (Figure S1) were used to amplify a 1028 bp upstream DNA segment (U) including 90 bp from the 5′ end of the coding sequence. This was inserted into the pGEM-T Easy vector (Promega) to give pGEM-U. Primers Up Δ*ac*/R & Low Δ*ac*/R (Figure S1) were used to amplify a 1031 bp downstream DNA segment (D) including 116 bp from the 3′ end of the coding sequence. This was inserted into the pGEM-T vector (Promega) to give pGEM-D. The insert in pGEM-U was then excised with *Not*I and *Spe*I and inserted into pGEM-D, cleaved with the same enzymes to yield pGEM-U-D. The kanamycin resistance (Km^R^) cassette of plasmid pHP45 Ω-Km was excised as a 2170 bp *Hin*dIII fragment and ligated with *Hin*dIII-digested pGEM-U-D to yield pGEM-U-Km-D. Finally, the U-Km-D deletion cassette was released from this plasmid by digestion with *Bam*HI & *Xba*I and inserted into *Bam*HI & *Xba*I-cleaved pPR27 [21]. This resulting construct was employed to transform *M. smegmatis* mc^2^155, yielding strain ΔAC in which a central ca. 900 bp segment of the MSMEG_4279 coding sequence has been replaced with a Km^R^ marker.

### Recombinant Rpf Isolation and Purification

A truncated form of *M. luteus* Rpf (RpfSm) was used for the analysis. The truncated protein comprises residues 42–134 of the native protein, encoding the conserved Rpf domain and an additional 20 amino acids downstream. It lacks the N-terminal signal sequence and the C-terminal LysM domain. Isolation and purification of the truncated form of *M. luteus* Rpf was as described previously [19].

### Statistical Analysis

Student’s test assuming unequal variance was performed for estimation of significance for comparative data. P-values are indicated as follows: * = p<0,05, ** = p<0,01, *** = p<0,001. ANOVA was applied to demonstrate significant difference in gene expression analysis by RT PCR. All data are presented as mean+\− standard error of the mean.

## Results

### Effect of Lipids on the Reactivation of Dormant Mycobacterial Cells

**Figure 2:**
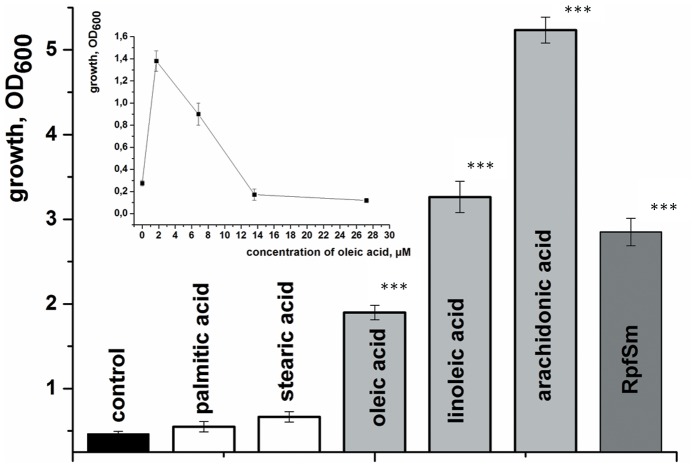
Fatty acid-induced resuscitation *of M. smegmatis* NC cells. NC cells were resuspended in reactivation medium to an initial OD_600_ ∼ 0.3 and resuscitated in batch format. The OD_600_ was measured after 5 d of resuscitation. Palmitic, stearic, oleic, linoleic and arachidonic acids were added at their optimum concentrations (4 µM, 4 µM 3.5 µM, 1.7 µM and 1.6 µM, respectively). The insert shows the concentration-dependence of oleic acid-mediated resuscitation. Each point represents the OD_600_ measurement after 5 d of resuscitation. This experiment was repeated three times with similar results; the error bars represent the standard error of the mean. Asterisks indicate that the results are significantly different from the control by Student’s t-test.

**Figure 3:**
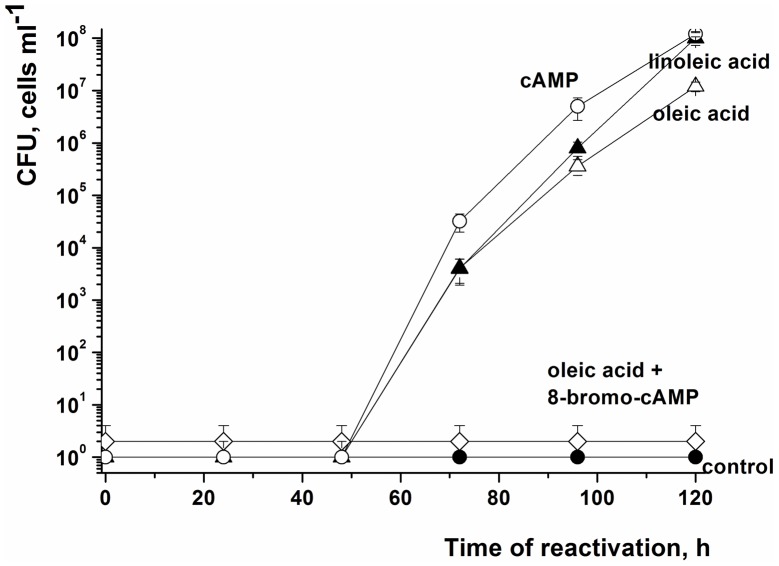
cAMP stimulates the resuscitation of *M. smegmatis* NC cells. NC cells were obtained and resuscitated in batch format. The OD_600_ was measured after 5 d of resuscitation. The concentrations of oleic acid, linoleic acid, cAMP and 8-bromo-cAMP were 2 µM, 1.7 µM, 3 mM and 2 mM, respectively. This experiment was repeated three times with similar results; the error bars represent the standard error of the mean.

**Figure 4:**
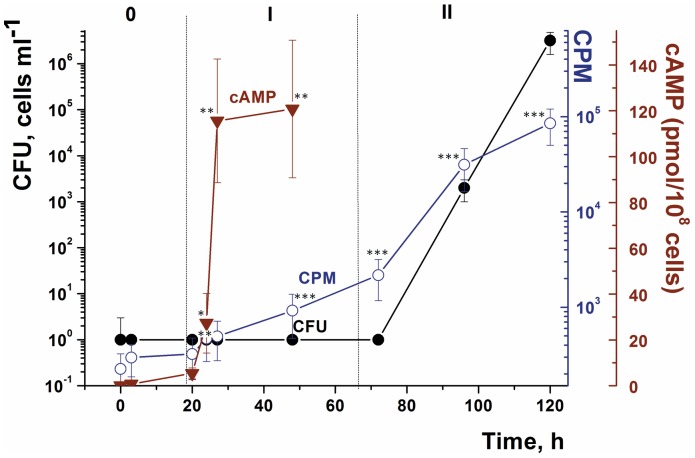
Comparison of the effects of linoleic acid and the uncoupler CCCP on the resuscitation of *M. smegmatis* NC cells. The resuscitation of NC cells was performed in MPN format. Each dilution was supplemented with linoleic acid or CCCP at the concentrations indicated. The ordinate shows the number of potentially viable (resuscitated) cells per ml of the initial NC population. This experiment was repeated twice with similar results; the error bars represent the standard error of the mean. Asterisks indicate that the result is significantly different from the control by Student’s t-test.

We used NC cells of *M. smegmatis* obtained from stationary phase cultures after growth under potassium limitation to search for low molecular weight lipids that induce resuscitation. Such cells were unable to multiply on solid medium (CFU = 0). However, they could be recovered in liquid medium containing either recombinant Rpf or SN taken from growing bacteria [15]. For the numerical estimation of potentially viable (recoverable) cells in starved populations, Rpf or SN was added to serially diluted cultures and the MPN (Most Probable Number) assay was employed [4], [15], [16]. Initially we found that exogenously added phospholipids resuscitated NC cells of *M. smegmatis* [22]. Chemically different phospholipids had similar activity [22] and we hypothesized that the observed resuscitation might be caused by free fatty acids liberated by the action of esterases and phospholipases found on the mycobacterial cell surface [23]. We therefore determined whether free fatty acids (FAs) have activity when added to the resuscitation medium. These experiments demonstrated that FAs with one or more unsaturated bonds do indeed stimulate the resuscitation of bacterial cells in a similar fashion to Rpf (Figures 2 & 3). Activity was concentration-dependent with an optimum of 2–10 µM for FAs (typical data for oleic acid are shown in the insert in Figure 2). The addition of 1.5 µM linoleic acid to the resuscitation medium led to the recovery of ca. 10^5^ cells/ml, compared with only 10^3^ cells/ml in the control sample (Figure 4). Because FAs are known to be effective uncouplers of oxidative phosphorylation in bacterial cells [24], we performed resuscitation experiments with the uncoupler, CCCP that is active in *M. smegmatis* [25]. These experiments demonstrated that CCCP is unable to stimulate resuscitation over a broad concentration range up to 1 µM (the maximum CCCP concentration that did not influence the growth of viable bacteria – Figure 4). This experiment rules out a possible role of the uncoupling effect of free fatty acids in resuscitation. Taking into account the fact that FAs were active at extremely low concentrations, a signaling role in resuscitation seems plausible.

### Involvement of cAMP in Resuscitation

In searching for a possible pathway that might be involved in sensing exogenous FAs, we noted that the product of the *M. tuberculosis rv2212* gene is a soluble adenylyl cyclase (AC), whose activity is stimulated by oleic and linoleic acids leading to increased cAMP production [26]. The *M. smegmatis* homologue is the product of gene MSMEG_4279 (66% aa identity with Rv2212). We hypothesized that the AC encoded by MSMEG_4279 might be involved in signal transmission in *M. smegmatis*, in which case resuscitation would be induced by elevation of the intracellular concentration of cAMP. To test this we added cAMP to the resuscitation medium at concentration of 3 mM, which is high enough to penetrate into cells of *Streptomyces coelicolor* [27] and *M. smegmatis* [28]. This produced a similar resuscitation effect to that previously observed following treatment with FAs (Figure 3). Increasing cAMP concentrations up to 10 mM did not alter the resuscitation effect (Figure S2). Dibutyryl cAMP is a more hydrophobic form of cAMP that penetrates into cells more readily and this was active at lower concentrations (0.5–1 mM) (Figure S2).

**Figure 5:**
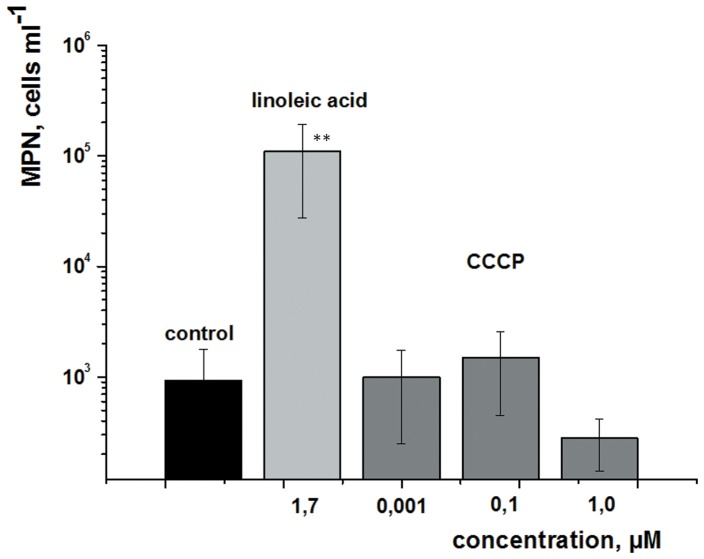
Intracellular cAMP levels and ^3^H-uracil incorporation during oleic acid-induced resuscitation of *M. smegmatis* NC cells. NC cells were obtained and resuscitated in batch mode. Oleic acid was added at a concentration of 3.5 µM. The intracellular level of cAMP was estimated after cells had been harvested and disrupted as described in the Materials and Methods. For samples taken during the first 48 h of resuscitation, metabolic activity was determined using ^3^H-uracil incorporation (denoted CPM on the Figure axis) as detailed in Materials and Methods. Dotted lines divide the overall process into three phases: 0 - true lag, I - metabolic activation, II - cell multiplication. This experiment was repeated three times with similar results. Error bars represent the standard error of the mean. Asterisks indicate that the results are significantly different from the values at zero time by Student’s t-test.

More detailed investigation showed that one day after the onset of oleic acid-mediated resuscitation, the intracellular concentration of cAMP dramatically increased from 0–10 to 120–130 pmol per 10^8^ cells (Figure 5). This increase was followed by the gradual resumption of cellular metabolism as judged by radioactive uracil incorporation, but cell multiplication did not commence until 72 h after the initial contact with oleic acid (Figure 5). The addition of 8-bromo-cAMP (a known inhibitor of AC [29]) to the reactivation medium completely abolished the resuscitation of NC cells (Figure 3) indicating the involvement of an AC in the process. Cells incubated in the resuscitation medium without added FA showed low levels of intracellular cAMP over the entire resuscitation period (Figure S3).

**Figure 6:**
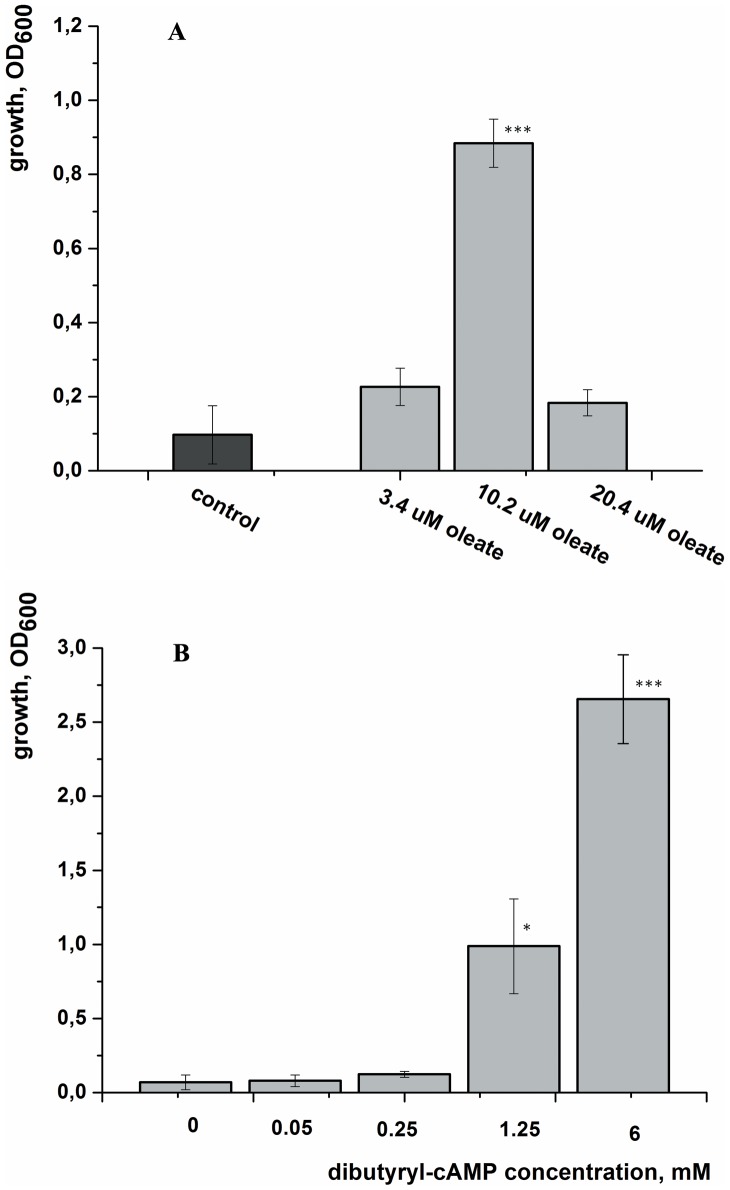
Fatty acid- and dibutyryl-cAMP-induced resuscitation *of M. tuberculosis* NC cells. NC cells were obtained inoculated to an initial OD_600_ = 0.2 and resuscitated in batch format. The OD_600_ was measured after resuscitation for 20 d (A) or 25 d (B). This experiment was repeated two times with similar results; the error bars represent the standard error of the mean. Asterisks indicate that the results are significantly different from the control by Student’s t-test.

Similar resuscitation experiments were performed with NC cells of *M. tuberculosis* obtained after adaptation to gradual acidification of the growth medium. Despite the fact that these cultures contained zero CFU, cells could be recovered in liquid medium containing SN taken from growing bacteria [16]. As with *M. smegmatis,* the resuscitation of NC cells of *M. tuberculosis* was induced by externally added oleic acid (optimal concentration ca. 10 µM) or by dibutyryl-cAMP (Figure 6). For these experiments, the growth medium contained tyloxapol instead of Tween-80. In contrast to *M. smegmatis* cells, cAMP was not active for NC *M. tuberculosis* cells presumably due to its poor penetration through the cell envelope.

**Figure 7:**
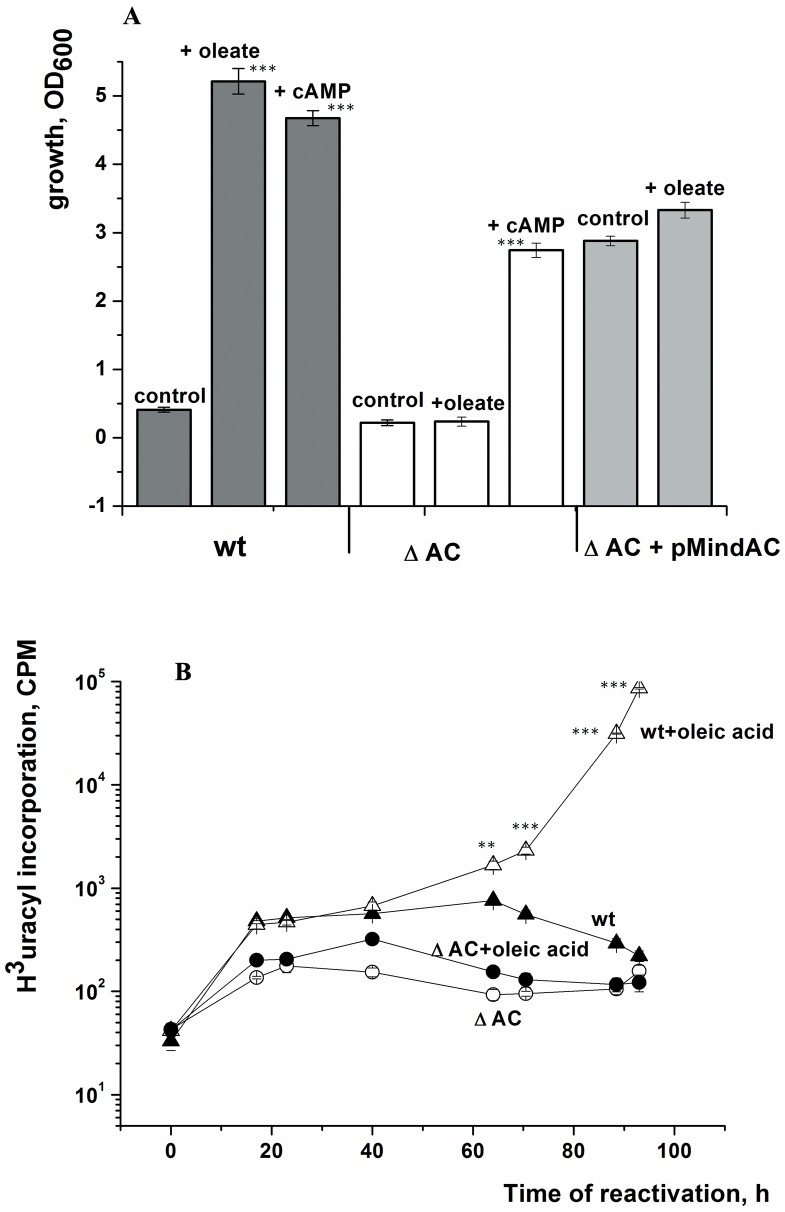
Involvement of the adenylyl cyclase encoded by MSMEG_4279 in the oleic acid-induced resuscitation of NC cells. NC cells were obtained and resuscitated in batch mode. In part A, the OD_600_ was measured after resuscitation for 5 d for the wild type and the complemented strain, ΔAC(pMindAc), and after 11 d for the ΔAC knock-out strain. Oleic acid and cAMP were added at concentrations of 3.5 µM and 3 mM, respectively. In part B, samples were taken at intervals over the first four days from cultures of the wild type and the mutant, both with and without oleic acid, for the estimation of cellular metabolic activity (uracil incorporation). Asterisks in Fig. 6A indicate that the results are significantly different from the control for each strain by Student’s t-test. Asterisks in Fig. 6B indicate significant difference between wt cells and wt+oleic acid by Student’s t-test.

To confirm the role of AC, a ΔAC mutant of *M. smegmatis* was constructed in which the MSMEG_4279 gene was inactivated by deleting most of the coding sequence (see Material and Methods). The ΔAC mutant was able to form NC cells, but they were unable to resuscitate in the presence of FAs as measured by growth stimulation or activation of metabolism (uracil incorporation) (Figures 7A and B). In the ΔAC mutant, intracellular levels of cAMP remained very low both with (in contrast to the wild type) and without (similar to the wild type) oleic acid addition (Figure S3). However, NC cells of the ΔAC mutant did reactivate in response to the exogenous addition of cAMP (3 mM) (Figure 7A) albeit at a rate somewhat slower than that observed for the wild type (visible growth occurred after 11 d of incubation as compared with 5 d for the wild type – data not shown).

The resuscitation defect of the ΔAC mutant was complemented by the introduction of a plasmid-encoded copy of the MSMEG_4279 gene. NC cells of the complemented strain, ΔAC(pMind-AC), resuscitated even in the absence of FA and the inducer, tetracycline (Figure 7A), presumably due to weak residual expression from the Tet-induced promoter in the absence of inducer (see also Figure 1). Interestingly, strain WT(pMind-AC), i.e. the wild type *M. smegmatis* strain hyper-expressing MSMEG_4279, did not develop NC cells under conditions when the wild type and the ΔAC strain did so (Figure 1). This correlated with a substantial increase of the intracellular cAMP concentration in the WT(pMind-AC) strain as compared with the WT(pMind) control during the transition of cells to the NC state in post-stationary phase (Figure 1). A similar result was obtained for the *M. tuberculosis* strain hyper-expressing MSMEG_4279 under conditions when the wild type developed a NC state in response to gradual acidification during prolonged stationary phase (Figure S4).

### Does FA-dependent Resuscitation Involve the Rpf Proteins?

**Figure 8:**
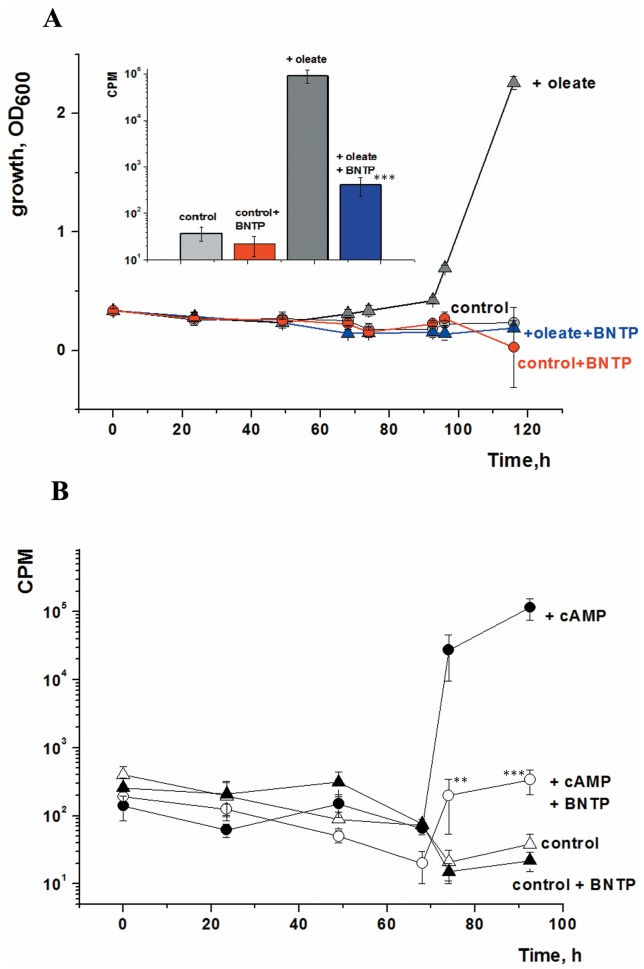
Oleic acid-mediated resuscitation of *M. smegmatis* NC cells is Rpf-dependent. NC cells were resuscitated in batch mode in the presence and the absence of both oleic acid (3.5 µM) (A) or cAMP (3.0 mM) (B) and the Rpf inhibitor, BNPT (1 µg/ml) in all four possible combinations. BNPT was added at zero time and again after 48 h of incubation. Samples were withdrawn periodically for OD_600_ determination (A) and estimation of metabolic activity using ^3^H-uracil incorporation (B) The insert to part A shows the level of uracil incorporation in the four cultures measured after 92 h of incubation. The error bars represent the standard error of the mean. Asterisks indicate significance between ^3^H-uracil incorporation by cells incubated in the presence of oleate vs both oleate and BNTP (A, insert) or in the presence of cAMP vs both cAMP and BNTP (B) by Student’s t-test.

Proteins of the Rpf family are known to control the reactivation of dormant and NC mycobacteria [10] and it was therefore important to determine whether FA- and Rpf-dependent resuscitation are somehow connected. To answer this question we performed resuscitation experiments with FA in the presence of 4-benzoyl-2-nitrophenylthiocyanate (BNPT). This compound, like other 2-nitrophenyl-thiocyanates inhibits the Rpf-dependent resuscitation of *M. smegmatis* NC cells [19]. BNPT inhibited the oleic acid-induced resuscitation and growth of NC cells of *M. smegmatis* (Figure 8A). A similar effect of BNPT was found when resuscitation was induced by cAMP (Figure 8B). It is important to note that in these experiments BNTP was applied at concentrations that did not affect the growth of viable *M. smegmatis* cells [19]. This experiment demonstrates that FA-induced resuscitation of NC cells of *M. smegmatis* is Rpf-dependent.

Significantly, NC cells incubated in the presence of BNTP showed increased uracil incorporation (see insert to Figure 8A). Normalisation of uracil incorporation per viable cell gives values of 900–1000 cpm per 10^3^ cells for actively dividing organisms in exponential cultures and 1500–2000 cpm per 10^3^ viable cells present after resuscitation of NC cells for 92 h (the viable cell count was estimated using the MPN assay). The similarity of the two values indicates that the provision of oleic acid results in the activation of metabolism in dormant cells, whilst the inhibition of Rpf activity prevents their multiplication.

**Figure 9:**
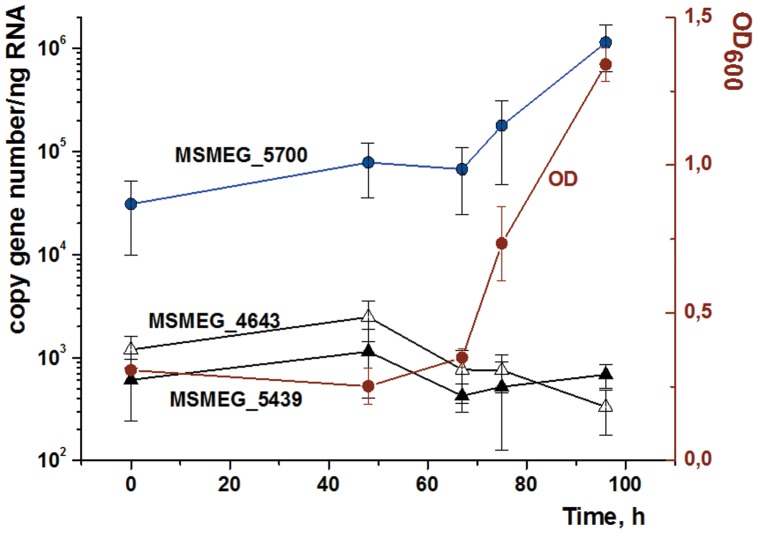
Expression profiles of three *M. smegmatis rpf* genes during oleic acid-mediated resuscitation of NC cells. NC cells were resuscitated in batch mode. Oleic acid was added at a concentration of 3.5 µM and the initial culture density was adjusted to give an OD_600_ = 0.3. RNA was isolated from cells withdrawn from the culture at different time points. Quantitative RT-PCR was performed using equal amounts of RNA (50 ng) as described in Materials and Methods. Each point is the mean of nine measurements (three technical replicates of the three biological replicates). The average OD_600_ values of the three replicate cultures are also shown. Error bars represent the standard error of the mean. Significance between expression level of MSMEG_5700 after 67 h of incubation was demonstrated by ANOVA (P<0,05).

Finally, we determined whether the elevated intracellular cAMP concentration that results from exogenous FA administration is accompanied by elevated *rpf* gene expression during the resuscitation period (in lag phase). For this RT-PCR study, we used equal amounts of total RNA isolated from cells sampled at different times of resuscitation. As expected, the amount of RNA isolated from NC bacteria was much lower than that obtained from actively multiplying cells. Under our resuscitation conditions we found that the expression levels of three of the four *rpf* genes of *M. smegmatis* did not change substantially for the first 48 h of incubation. Between 48 h and 67 h the expression level of MSMEG_5700 (*rpfA*) did not change, whereas MSMEG_5439 (*rpfB*) and MSMEG_4643 (*rpfF*) were slightly down regulated (Figure 9). After 67 h, in early log phase, *rpfA* exhibited significant up-regulation whereas the expression levels of *rpfB* and *rpfF* showed little change (Figure 9). The fourth *rpf* gene (MSMEG_4640) found in this organism could not be monitored because we were unable to design specific primers for this gene. In the control culture (no FA added) copy numbers of the *rpfA*, *rpfB* and *rpfF* genes remained constant throughout the experiment (data not shown). This experiment, along with the results obtained following the application of BNPT, demonstrates that the FA-stimulated resuscitation process requires Rpf for the successful growth.

## Discussion

In the present study we found that exogenously added free fatty acids induce the resuscitation of dormant, NC *M. smegmatis* cells obtained in stationary phase after cultivation under potassium-limiting conditions. Zhang at al. have previously reported the reactivation of starved *M. tuberculosis* cells in the presence of phospholipids [14]. The active compounds in their experiments could also have been fatty acids derived from phospholipids by esterases and phospholipases. However, this suggestion is not consistent with the author’s findings that resuscitation activity was abolished after digestion of the phospholipids with phospholipase A_2_ [14]. This apparent discrepancy might arise because uncontrolled amounts of FAs would have been released after phospholipase A_2_ digestion of phospholipids in the previously published work. We have shown that an optimum FA concentration is needed to produce the observed resuscitation effect, higher concentrations being ineffective or even inhibitory (Figure 2). Alternative sources of FAs may be important in host environments. For example, Daniel at al. have shown that triacylglycerols (TAG) accumulate in hypoxic dormant-like *M. tuberculosis* cells both in *in vitro* [30] and in lipid-loaded macrophages [31]. We suggest that TAG could be used as a source of intracellular FAs after TAG hydrolysis at the onset of resuscitation. Interestingly, in the work of Daniel at al, free FAs of host origin are used for TAG formation in *M. tubercuclosis* cells during the adoption of a dormant state [31]. During this period cells are metabolically active; the FAs are used during the *transition* from the active to the dormant state, but not in the state of dormancy *per se* (when cells are not metabolically active, by definition). Therefore FAs may participate in both the formation of dormant cells (as metabolic substrates) and in their resuscitation (as a trigger).

The low active concentrations of FAs and their chemical specificity (i.e. superior activity of unsaturated FAs) suggested that they may play a signaling role in the resuscitation process. A nutritional role for FAs could be ruled out as the Tween-free reactivation medium contains all the compounds required for active bacterial growth and it is, in any case, sufficient for Rpf-dependent resuscitation [15].

FAs are known to participate in variety of signaling cascades in microorganisms. For example, arachidonic acid serves as chemo-attractant for *Dictyostelium discoideum* [32] and FAs serve as signals for the differentiation of *Myxococcus xanthus* [33]. Factor d2 isolated from number of Gram-positive bacteria contains an unsaturated FA which, at concentrations between 7–20 µM, promotes the growth of resting bacterial cells [34]. At a higher concentration, oleic acid induces the germination of *Entomophthora culicis* conidia [35].

In the present paper we establish, for the first time, that the resuscitation of NC mycobacterial cells is triggered by unsaturated FAs including arachidonic acid. The existence of an optimal concentration range for FA-mediated resuscitation activity (Figure 2, insert) could be explained by a toxic effect of unsaturated FAs, which provoke cytoplasmic membrane degradation at high concentrations [36]. For *M. smegmatis,* a toxic effect of oleic acid has been found at concentrations above 20 µM (M. Shleeva unpublished observation). At the same time, oleic acid (in the form of OADC supplement, or originating from the metabolism of Tween 80) is an important component in a number of media formulated for the successful initiation and optimal growth of mycobacteria [37]. Presumably, any possible negative effect of FAs in mycobacterial growth media is compensated by the presence of albumin, which effectively binds excess oleic acid. On the other hand, this binding could reduce the free oleic acid concentration to a level below that required for stimulating the resuscitation of NC cells (Figures 2 and 6A). Media supplemented with OADC (0.2 mM oleic acid and 60 mM BSA) may therefore require the addition of exogeneous stimulators, (e.g. Rpf proteins) in order to recover NC cells in the population [38].

In *M. tuberculosis* exogenous FAs are sensed by a cytosolic adenylyl cyclase encoded by *rv2212* [26]. The *M. smegmatis* homologue encoded by MSMEG_4279 was a likely potential candidate for causing the increased intracellular level of cAMP observed following the addition of FA to NC *M. smegmatis* cells (Figure 5). An increased intracellular cAMP content was reported when the effects of ADC and OADC (oleic acid, albumin, dextrose, catalase) supplements on cAMP levels in *M. bovis* were compared [39]. Two lines of evidence support the involvement of the AC encoded by MSMEG_4279 in the observed reactivation of NC cells by FAs. First, FA could be substituted by cAMP in resuscitation experiments and second, the MSMEG_4279 knock-out mutant (ΔAC) was unable to resuscitate in the presence of FA but did resuscitate in the presence of cAMP. The hyper-expression of the MSMEG_4279 gene in wild type *M. smegmatis* cells prevented the transition of viable cells to dormant, non-culturable forms, probably due to the presence of unnaturally high levels of intracellular cAMP (Figure 1), which did not allow the cells to become dormant. Complemented cells of the *M. smegmatis* adenylyl cyclase mutant, ΔAC(pMind-AC) with uncontrolled expression of MSMEG_4279 showed properties intermediate between those of the wild type and the ΔAC strain: such cells were able to produce NC cells but they also resuscitated from the NC state without any external stimulus (Figure 7A). It is interesting that the stimulatory effect of FA on Rv2212 in *M. tuberculosis* is only seen at low intracellular ATP concentrations [26] similar to those found in dormant, NC *M. smegmatis* cells [40]. Indeed, the addition of FAs to actively growing *M. smegmatis* cells at concentrations used for resuscitation did not stimulate bacterial growth (M. Shleeva, unpublished). Similarly with *M.smegmatis*, oleic acid and dibutyryl cAMP cause reactivation of dormant *M.tuberculosis* cells (Figure 6A, B). Also hyper-expression of the MSMEG_4279 gene in *M.tuberculosis* cells prevented transition of viable cells to dormant, non-culturable forms (Figure S4). This allows us to suggest that the mechanism of FA-depependent resuscitation of dormant *M.tuberculosis* could be similar to *M.smegmatis* however, participation of *M.tuberculosis* AC in this process needs to be clarified.

The pleiotropic effects of cAMP, which acts as a second messenger in a wide variety of bacterial processes, is well known [41]. The involvement of cAMP and AC in the exit from constitutive dormancy (spore germination) is established [27]. The cAMP content of spores of streptomyces is very low. However, the cAMP level substantially increases during spore germination and then decreases again during the later phase of mycelial growth [27], [42]. An AC mutant of *Str. coelicolor* showed defective spore germination and this phenotype was suppressed by the addition of cAMP at concentrations above 1 mM [27]. This mutant also exhibited morphological changes in colonies growing on the surface of agar [27]. In later work, the cAMP receptor in *Str. coelicolor*, which is homologous to the *E. coli* Crp protein, was found. Crp knock-out mutants exhibited similar defects in spore germination and other physiological effects as those observed in AC mutants. These findings led to the conclusion that the cAMP-AC-CRP system plays an important role in the control of spore germination in *Str. coelicolor* [43]. In contrast to *Streptomyces*, spores of the social amoeba *Dictyostelium discoideum* contain ca. 10 times more cAMP than amoeboid cells. The high level of cAMP decreased only after spore germination. However, despite the high level of cAMP at the onset of spore germination its intracellular concentration transiently increased in the early germination phase [29], which is similar to what happens during the germination of *Streptomyces* spores.

The downstream processes that link increased cAMP concentrations to cell resuscitation are not yet clear. In *M. tuberculosis*, two cAMP-associated transcriptional factors CRPmt and Cmr are well characterized [44]. Both factors may regulate a number of biologically important pathways including respiration and fatty acid & carbohydrate metabolism [44]. Bai et al proposed a putative CRPtb regulon that includes over 100 genes, a substantial number of which are also involved in the control of dormancy in *M. tuberculosis* [45]. In the context of this investigation it is significant that CRPtb (Rv3676) activates the expression of only one of the five *rpf* genes (*rpfA*) found in *M. tuberculosis* [46]. Significantly, of the three *rpf* genes monitored in this investigation, *rpfA* was the only one to show enhanced expression during resuscitation. Rickman et al [2005] suggested that Rv3676 may control the reactivation of dormant cells despite the fact that cAMP did not enhance Rv3676 binding at the *rpfA* promoter [46]. It is interesting that in *Corynebacterium glutamicum*, the *rpf2* gene (a homologue of *M. tuberculosis rpfB*) is also under the positive control of the cAMP-dependent GlxR transcriptional regulator, which is a homologue of Rv3676. In contrast to Rv3676 in *M. tuberculosis*, cAMP enhanced the binding of *C. glutamicum* ClxR to the *rpf2* promoter. However, hyper-expression of ClxR resulted in the up-regulation of *rpf2* expression when bacteria were grown on acetate but not when they were grown on glucose [47].

From the above discussion, it is evident that any possible link between cAMP levels and the induction of *rpf* gene expression in the resuscitation phase is far from simple. In our experiments, we found that addition of the Rpf inhibitor (BNPT) prevents the resuscitation of NC *M. smegmatis* cells in the presence of oleic acid (Figure 8A). This experiment supports the involvement of Rpf in FA-mediated resuscitation. The observed up-regulation of *rpfA* transcription (Figure 9) adds additional support to a possible causative link between AC activation and the expression of at least one of the Rpf proteins probably via one of two homologues of Rv3676 in *M. smegmatis* (MSMEG_6189, 97% identity, or MSMEG_0539, 91% identity). However, up-regulation of *rpfA* expression was delayed until the beginning of cell multiplication, which occurred long after the time when the intracellular level of cAMP increased (Figures 5 and 9). Because up-regulation of *rpfA* did not precede start of cell multiplication, RpfA may therefore control acceleration of cell multiplication by Rpf –dependent cell wall remodeling [10] in early log phase as a late event (after 67 h, Figure 9) allowing successful culture growth. Indeed, upon resuscitation without inducers, *M.smegmatis* NC cells are able to make several generations but further development stopped (Shleeva, personal communication). Thus, the resuscitation pathway may be separated into three phases as shown in Figure 5: true lag phase (0), metabolic reactivation (I) and cell multiplication (II). Indeed, secretion of Rpf proteins in *M. smegmatis* batch cultures was correlated with active growth but not with lag phase [15]. At the same time, FAs mediate their effect via cAMP during the initial stages of this process, resulting in the activation of cellular metabolism (metabolic reactivation) as evidenced by the kinetics of uracil incorporation (phase I, Figure 5) and their role does not seem to be connected directly to Rpf activity (Figure 9). However, we cannot exclude the possibility that cAMP may control *rpf* expression at an earlier stage of the resuscitation process because there is significant heterogeneity within dormant cell populations. The fraction of resuscitable cells in the population did not exceed a few percent; the number of resuscitable cells in the presence of FA was ca. 10^5^ per ml (Figure 4), whereas the total cell count was ca. 10^8^ cells per ml (not shown). The presence of unresponsive cells containing RNA may mask any measurable change in gene expression (measured at the population level) until the proportion of viable cells reaches some threshold level.

Previously published results on the regulation of *rpf* gene expression during the resuscitation of NC or dormant mycobacteria are rather controversial. It was reported that the expression of four of the five *M. tuberculosis rpf* genes (*rpfA, rpfB, rpfD and rpfE*) was down-regulated during the reactivation of a persistent infection in mice [48]. On the other hand, Gupta et al [49] reported a significant transient up-regulation of the expression of *M. tuberculosis rpfA* and *rpfD* during the resuscitation of cells from a NC state *in vitro* [49]. The absence of a detailed description of their resuscitation experiment (e.g. viability was not monitored by MPN/cfu) makes it difficult to compare these results with ours.

Evidently, cell metabolic activity is also restored when fresh medium (without any specific stimulators) is provided, as adjudged by uracil incorporation, (Figure 7B). However, it does not lead to a visible growth, probably because Rpf synthesis remains at a low level, according to real-time PCR measurements. Indeed, Rpf-dependent resuscitation *in vitro* was shown in experiments in which NC *M. smegmatis* cells were resuscitated in a FA- and Tween-free medium in the presence of Rpf [15]. Similarly, NC *M. smegmatis* cells hyper-expressing Rpf were able to resuscitate spontaneously without any additions [15].

In conclusion, the present study has revealed a new resuscitation pathway in which free fatty acids play the role of the initial inducer. Because mycobacteria contain a number of ACs that respond to different environmental stimuli [44], we cannot exclude the possibility that other distinct “dormancy breaking” signals may also exist [17]. Because lipids are evidently important nutrients for *M. tuberculosis* during growth in animal tissues [50], [51], the observed resuscitation activity of FA could mean that in particular host environments *in vivo*, where FA concentrations are appropriate, mycobacterial cells continually receive “resuscitation-inducing signals” that do not allow them to adopt a truly dormant state.

## Supporting Information

Figure S1 **Primers used.**
(PDF)

Figure S2 **Concentration dependence of cAMP or dibutyryl cAMP-mediated resuscitation.** NC cells were obtained and resuscitated in batch format. The OD_600_ was measured after 5 d of resuscitation. Different concentration (0–10 mM) of cAMP or dibutyryl cAMP were added in the onset of resuscitation. This experiment was repeated two times with similar results; the error bars represent the standard error of the mean.
(TIF)

Figure S3 **Intracellular cAMP levels during oleic acid-induced resuscitation of** ***M. smegmatis*** **NC cells of wild type and ΔAC strains.** NC cells were obtained and resuscitated in batch mode. Oleic acid was added at a concentration of 3.5 µM. The intracellular level of cAMP was estimated after cells had been harvested and disrupted as described in the Materials and Methods. The error bars represent the standard error of the mean. Asterisks indicate significant difference between wt cells and wt+oleic acid by Student’s t-test.
(TIF)

Figure S4 **Hyper-expression of the MSMEG_4279 adenylyl cyclase abolishes the transition of** ***M.tuberculosis*** **cells to the NC state.** *M. tuberculosis* cells were grown in Sauton medium under conditions described in Materials and Method. Strains harbouring pMind (empty plasmid vector) or pMindAc (adenylyl cyclase hyper-expression plasmid) are denoted with circles and triangles, respectively. Samples were withdrawn periodically for CFU determination.
(TIF)
