# Correction: Cyclic Amp-Dependent Resuscitation of Dormant Mycobacteria by Exogenous Free Fatty Acids

**DOI:** 10.1371/journal.pone.0097206

**Published:** 2014-05-02

**Authors:** The PLOS ONE Staff

The images for Figures 4 and 5 are incorrectly switched. The image that appears as Figure 4 should be Figure 5, and the image that appears for Figure 5 should be Figure 4. The figure legends appear in the correct order. The author has provided correct versions of Figures 4-5, which can be viewed below.

**Figure 4:**
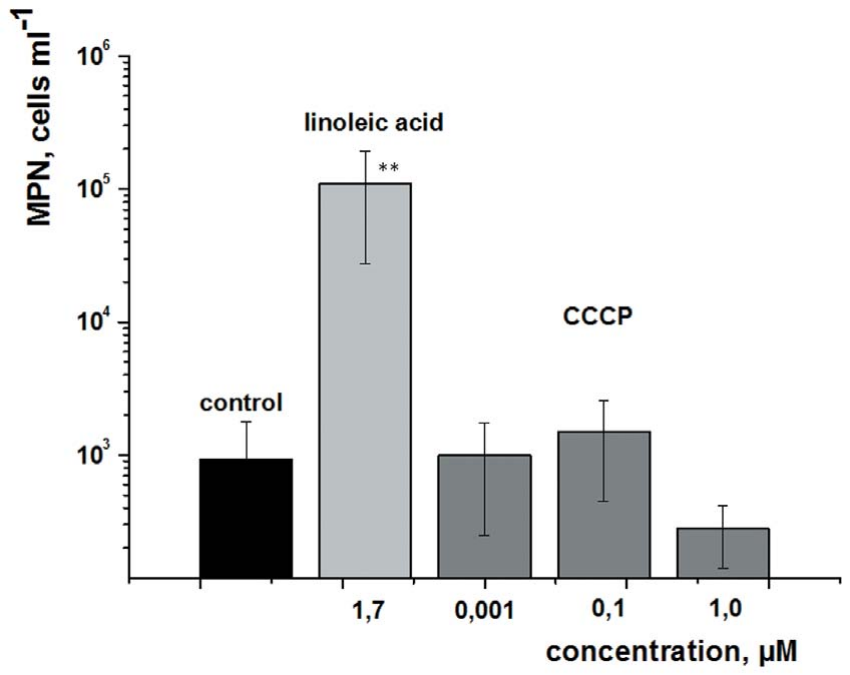
Comparison of the effects of linoleic acid and the uncoupler CCCP on the resuscitation of *M. smegmatis* NC cells. The resuscitation of NC cells was performed in MPN format. Each dilution was supplemented with linoleic acid or CCCP at the concentrations indicated. The ordinate shows the number of potentially viable (resuscitated) cells per ml of the initial NC population. This experiment was repeated twice with similar results; the error bars represent the standard error of the mean. Asterisks indicate that the result is significantly different from the control by Student’s t-test.

**Figure 5:**
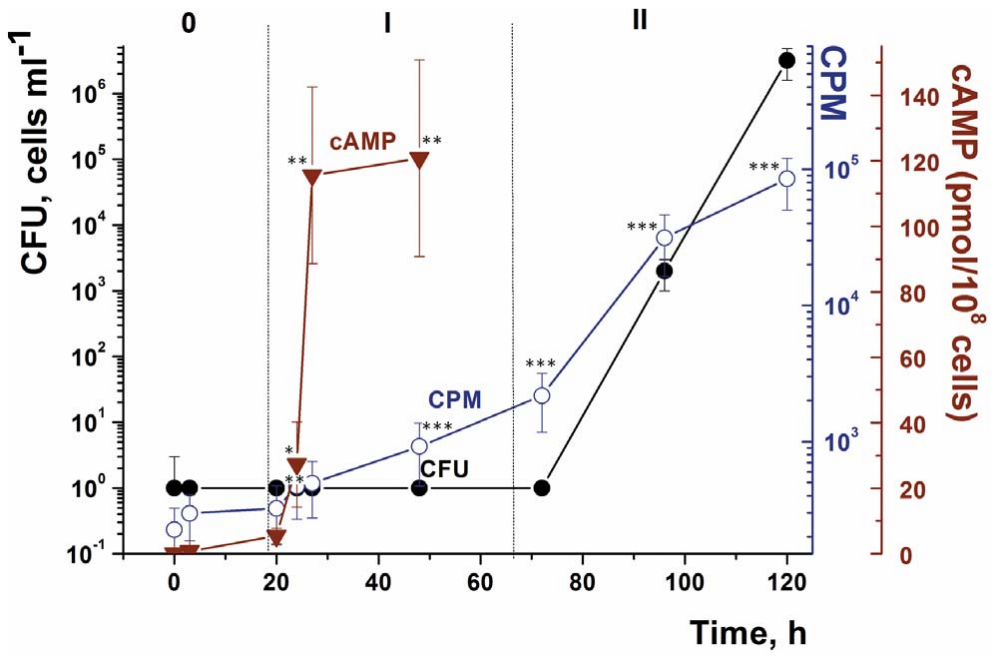
Intracellular cAMP levels and ^3^H-uracil incorporation during oleic acid-induced resuscitation of *M. smegmatis* NC cells. NC cells were obtained and resuscitated in batch mode. Oleic acid was added at a concentration of 3.5 µM. The intracellular level of cAMP was estimated after cells had been harvested and disrupted as described in the Materials and Methods. For samples taken during the first 48 h of resuscitation, metabolic activity was determined using ^3^H-uracil incorporation (denoted CPM on the Figure axis) as detailed in Materials and Methods. Dotted lines divide the overall process into three phases: 0 - true lag, I - metabolic activation, II - cell multiplication. This experiment was repeated three times with similar results. Error bars represent the standard error of the mean. Asterisks indicate that the results are significantly different from the values at zero time by Student’s t-test.

## References

[1] Shleeva M, Goncharenko A, Kudykina Y, Young D, Young M, et al (2013) Cyclic Amp-Dependent Resuscitation of Dormant Mycobacteria by Exogenous Free Fatty Acids. PLoS ONE 8(12): e82914 doi:10.1371/journal.pone.0082914 24376605 10.1371/journal.pone.0082914 PMC3871856

