# Supplementary material for: Cyclic Amp-Dependent Resuscitation of Dormant Mycobacteria by Exogenous Free Fatty Acids
Source: PLoS One. 2013 Dec 23;8(12):e82914. doi: 10.1371/journal.pone.0082914 (PMC3871856; doi:10.1371/journal.pone.0082914)
Supplement: Figure S1 — Primers used. (PDF) [file pone.0082914.s001.pdf]

Figure S1

Primers used for quantitative real-time PCR.

MSMEG_5700F (5’–CCTTTTGGCTCCTTGGGGTG–3’)

MSMEG_5700R (5’-GTCCACTCATAAGCGTCGAGG-3’)

MSMEG_5439BF (5’–CACAAGACCGTGACCCTCTC– 3’)

MSMEG_5439BR (5’–GCGATCAGAAACCTCGAAGCC– 3’)

MSMEG_4643F (5’–CCTTTCGTGCCTGACCGAGA– 3’)

MSMEG_4643R (5’–GATGTTCTTCACTTCGTTCCTTTC– 3’)

MSMEG_4640F (5’–GCATGGCCGAAGTGCGGTG– 3’)

MSMEG_4640R (5’–CAGGTCCAGGATGCCCAGG– 3’)

Primers used for cloning

Name	Sequence 5′ - 3′	Enzyme
Up pMind-Ac	CGGATCCTGCAGCAGCTCGTCACTCACCACA	*Bam*HI
Low pMind-Ac	CACTAGTGCGGGGCAACGACGAGCTGACGGT	*Spe*I
Up Δ*ac*/L	CGGATCCCGGCCCAGCGCCTTGAGA	*Bam*HI
Low Δ*ac*/L	GAAAGCTTACCGGTCGAGGTAGGAGATGA	*Hin*dIII
Up Δ*ac*/R	CAAGCTTGCGCGAGGCGCTGGGAGACT	*Hin*dIII
Low Δ*ac*/R	GTCTAGAGGTGCATGTTGGCTGCGGGCTTGAT	*Xba*I
