# Supplementary figures and images for: Cyclic Amp-Dependent Resuscitation of Dormant Mycobacteria by Exogenous Free Fatty Acids
Source: PLoS One. 2013 Dec 23;8(12):e82914. doi: 10.1371/journal.pone.0082914 (PMC3871856; doi:10.1371/journal.pone.0082914)

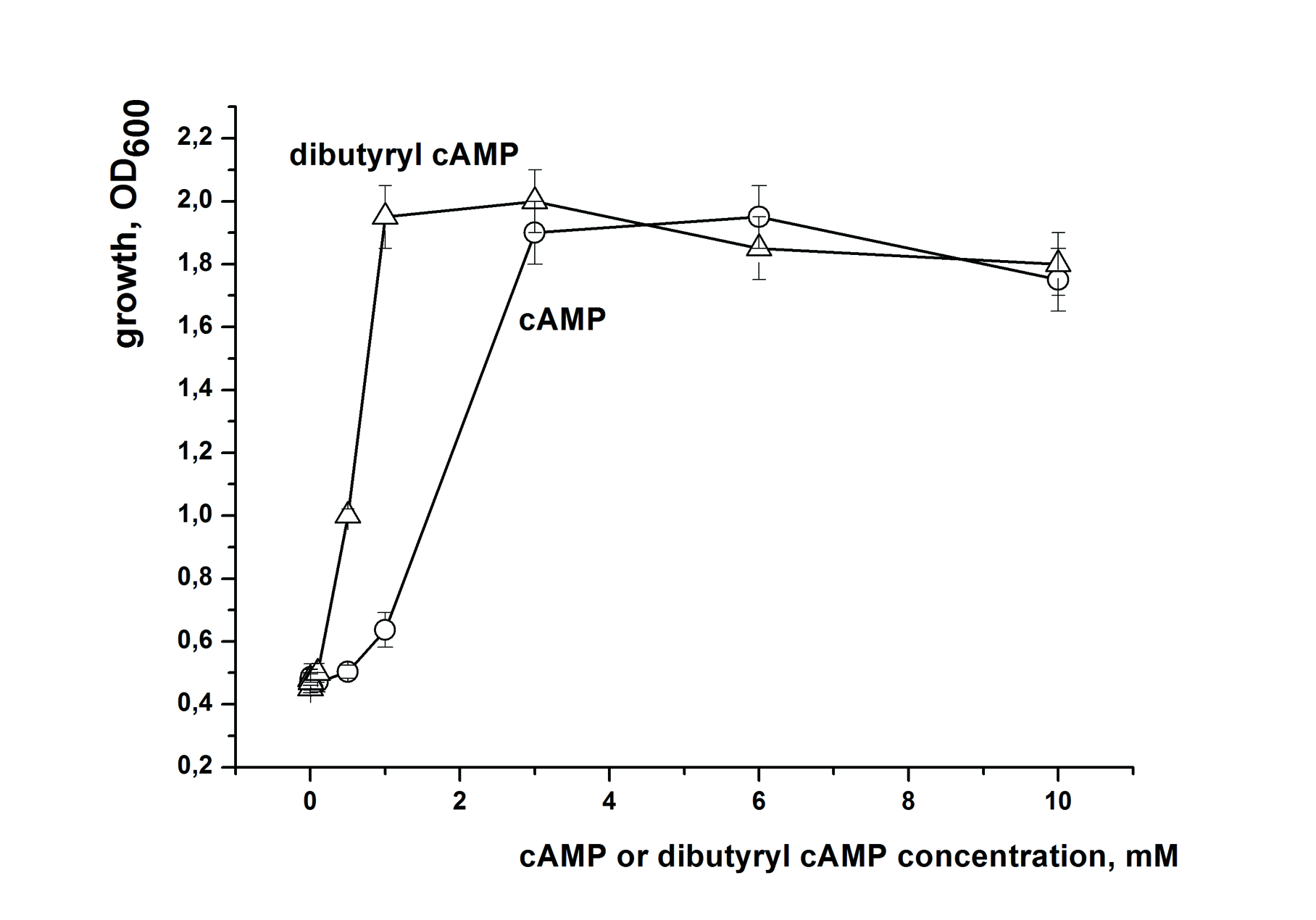

Supplement: Figure S2 — Concentration dependence of cAMP or dibutyryl cAMP-mediated resuscitation. NC cells were obtained and resuscitated in batch format. The OD600 was measured after 5 d of resuscitation. Different concentration (0–10 mM) of cAMP or dibutyryl cAMP were added in the onset of resuscitation. This experiment was repeated two times with similar results; the error bars represent the standard error of the mean. (TIF) [file pone.0082914.s002.tif]
